# A vision of human–AI collaboration for enhanced biological collection curation and research

**DOI:** 10.1093/biosci/biaf021

**Published:** 2025-03-28

**Authors:** Alan Stenhouse, Nicole Fisher, Brendan Lepschi, Alexander Schmidt-Lebuhn, Juanita Rodriguez, Federica Turco, Andrew Reeson, Cécile Paris, Peter H Thrall

**Affiliations:** National Collections and Marine Infrastructure Research Unit, Commonwealth Scientific and Industrial Research Organisation (CSIRO), Canberra, Australia; National Collections and Marine Infrastructure Research Unit, Commonwealth Scientific and Industrial Research Organisation (CSIRO), Canberra, Australia; National Collections and Marine Infrastructure Research Unit, Commonwealth Scientific and Industrial Research Organisation (CSIRO), Canberra, Australia; Parks Australia, Canberra, Australia; Centre for Australian National Biodiversity Research, Canberra; National Collections and Marine Infrastructure Research Unit, Commonwealth Scientific and Industrial Research Organisation (CSIRO), Canberra, Australia; Centre for Australian National Biodiversity Research, Canberra; National Collections and Marine Infrastructure Research Unit, Commonwealth Scientific and Industrial Research Organisation (CSIRO), Canberra, Australia; National Collections and Marine Infrastructure Research Unit, Commonwealth Scientific and Industrial Research Organisation (CSIRO), Canberra, Australia; CSIRO's Data61 Research Unit; CSIRO's Data61 Research Unit; National Collections and Marine Infrastructure Research Unit, Commonwealth Scientific and Industrial Research Organisation (CSIRO), Canberra, Australia

**Keywords:** human–AI interaction, natural history, digital curator, biodiversity, specimen data

## Abstract

Natural history collections play a crucial role in our understanding of biodiversity, informing research, management, and policy in areas such as biosecurity, conservation, climate change, and food security. However, the growing volume of specimens and associated data presents significant challenges for curation and management. By leveraging human–AI collaborations, we aim to transform the way biological collections are curated and managed, realizing their full potential in addressing global challenges. In this article, we discuss our vision for improving biological collections curation and management using human–AI collaboration. We explore the rationale behind this approach, the challenges faced in data management, general curation problems, and the potential benefits that could be derived from incorporating AI-based assistants in collection teams. Finally, we examine future possibilities for collaborations between human and digital curators and collection-based research.

In this article, we outline our vision for human–AI collaborative curation of natural history collections. We first discuss the current value and challenges of biological collections, highlighting the need for innovative curation and management. We then explore how AI-based digital curators could be developed and integrated into collection teams’ use, drawing on emerging AI technologies such as large language models (LLMs), multimodal AI, and agentic systems. Finally, we consider the steps needed to realize this vision and the transformative impact it could have on biodiversity research and global challenges.

Our aim is not to advocate for the replacement of human expertise, which remains essential for high-level decision-making, quality control, and advancing the theoretical frameworks of taxonomy and systematics. Instead, we envision AI-based digital curators assisting human curators and researchers by leveraging the complementary strengths and mitigating the weaknesses of each (Fischer 2023). These AI assistants could handle many routine tasks, identify anomalies for human review, and potentially suggest new hypotheses. This would allow people to focus on complex problems and scientific interpretation. Such collaborations have the potential to dramatically enhance curatorial capabilities and would ensure the vast knowledge contained in natural history collections is more accessible, integrated, and applied to critical issues in biodiversity conservation, climate change adaptation, and beyond.

## The value of biological collections

There are an estimated 2 billion–4 billion specimens in natural history collections around the world, less than 20% of which are easily discoverable or accessible (Johnson et al. 2023). These collections represent a critical but underused resource of global significance. Despite the currently limited visibility and availability of much of this fundamental biodiversity data, collection specimens already make significant contributions to efforts to address global challenges in areas ranging from ecology, evolution, conservation, and agriculture to human health and climate change (Funk 2018, Meineke et al. 2019). The intrinsic value of these collections is dependent on continued expansion in scope and diversity, especially through meticulous curation of specimen data. Their usefulness is significantly increased when both the specimens and their associated data adhere as closely as possible to FAIR data principles (i.e., findable, accessible, interoperable, and reusable; Wilkinson et al. 2016).

Importantly, the specimens contained in biological collections provide an irreplaceable historical record of our world in time and space. They are the basis for taxonomic and systematic research and contribute directly to our ability to document and characterize these fundamental aspects of biodiversity. Moreover, these collections can help us understand and answer questions about past, present, and future environmental states and are the physical basis for our understanding of the natural world and our place in it (Johnson et al. 2023). Not only are biological specimen collections critical resources for fundamental taxonomic research and species discovery (Bebber et al. 2010, Kawahara et al. 2023), but the records contained in these collections have been central to theories such as continental drift and, in recent decades, to the emergence of new fields such as evolutionary medicine (Funk 2018).

They are also key to better understanding ecological and evolutionary processes by determining phylogenetic relationships (Soltis and Soltis 2016) and through mapping species distribution and trait changes through time. This information can be used in applications in conservation and biodiversity, such as managing actions around threatened species, addressing biosecurity risks, or reducing impacts from invasive species (Chauvel et al. 2006, Greve et al. 2016, Beaulieu et al. 2018). Collection specimens are also vital for addressing issues affecting human health and society, such as assessing human impacts on biodiversity, disease origins and spread, climate change impacts and adaptations, and global food supply (Suarez and Tsutsui 2004, Funk 2018, Meineke et al. 2019, Thompson et al. 2021, Turbek et al. 2023). The information held in natural history collections is therefore of critical importance as we seek to understand, adapt to, and mitigate some of the problems resulting from the Anthropocene (Steffen et al. 2011, Meineke et al. 2019) and to ensure environmental and societal resiliency (National Academies of Sciences, Engineering, and Medicine 2020).

The physical specimens preserved in natural history collections are a fundamental resource that continues to increase in relevance, particularly as the environmental and societal problems we face become more acute. Many applications described above are only possible because of the development of new methods that allow us to more effectively mobilize and extract information from collection specimens. However, new technologies and techniques result in even more data that also requires curatorial resources. Examples include 3D scanning, computer tomography, advanced microscopy, and X-ray, infrared, multispectral, and hyperspectral imaging (Mizutani and Suzuki 2012, Keklikoglou et al. 2019, Théroux‐Rancourt et al. 2020). These offer opportunities for advanced image and feature analysis, such as phenomics and morphometrics (Borges et al. 2020, Lendemer et al. 2020, Pearson et al. 2020, Heberling 2022), without destructive sampling. Advances in genomic techniques include nondestructive DNA extraction from physical specimens and extraction even after long-term storage in solutions such as formalin (Appleyard et al. 2021, Hahn et al. 2022). Other high-throughput screening and analysis methods may accelerate the provision of molecular and genetic information for bioprospecting and biodiscovery (Mawalagedera et al. 2022) and support environmental, human health, and bioengineering applications, as well as novel research areas (Frisvold 2023).

Beyond new analytical techniques and maintaining curatorial efforts, fully realizing the potential of biological collections to contribute to fundamental science, as well as diverse national and global challenges, requires efficient at-scale mobilization and management of specimen data. It is increasingly recognized that digitization of collection specimens is a critical step in this process. Digital specimen data (e.g., high-resolution images) provide the foundation for advanced computational analysis using AI and machine learning (Greeff et al. 2022). These have the potential to enable rapid image processing, data extraction, and analysis, overcoming some limitations of manual examination, and are crucial for efficiently processing the immense volumes of data contained within the collections.

Recognition of the importance of digital data has meant that the imaging of natural history collections is being tackled by an increasing number of institutions around the world. Larger-scale national and international initiatives such as iDigBio (for *Integrated Digitized Biocollections*, www.idigbio.org/about/mission), DiSSCo (for *Distributed System of Scientific Collections*, www.dissco.eu), the Atlas of Living Australia (ALA; www.ala.org.au) and the Global Biodiversity Information Facility (GBIF; www.gbif.org) are aimed at building cross-institutional networks and data standards to efficiently share biodiversity data. As specimens are digitized and their data are made available globally, their potential contribution to current and future scientific research and education is magnified. However, there are multiple challenges that remain to be addressed before this can be successfully achieved.

## Challenges

Some of the challenges that biological collections face include the erosion of taxonomic and curatorial capability, reduced budgets and academic support (Bradley et al. 2014), the sheer volume of specimens and the associated data to be managed, and the growing recognition of the need to rapidly mobilize and efficiently coordinate and share the information held in natural history collections at global scales.

### Curatorial expertise and capacity

Physical specimens and data curation support our ability to make effective use of specimen-related information. For biological collections to help tackle emerging global issues, they need to be adequately resourced, with a trained and diverse workforce that has expertise in taxonomy, curation, data management, biodiversity science, and informatics. However, biological collections face significant constraints in terms of funding and the breadth of available taxonomic expertise, exacerbated worldwide by a limited and decreasing number of taxonomists with the skills needed to identify, describe, and catalogue taxa and specimens (Bradley et al. 2014, European Commission et al. 2022).

This often results in a backlog of specimens waiting to be curated, identified, and databased, especially as specimen collecting continues. There are in excess of an estimated 6 million species yet to be described (Liu et al. 2022), many of which may already exist in collections around the world (Greeff et al. 2022), although the majority of unknown species are still to be collected (Mora et al. 2011). Meanwhile, rapid changes to our ecosystems are undoubtedly resulting in the extinction of many species even before they are discovered or described (Tedesco et al. 2014).

Other curatorial and research challenges for physical collections include the creation of reliable digitized specimen data (high-quality images, genetic information, collection metadata), as well as the distribution of these data to relevant researchers and end users as they become available. Currently, one of the most time consuming and risky operations that physical collections undertake is the constant movement of specimens in and out of collections via loans between institutions. Any reduction in the need for specimens to be shipped around the world that results from increased mobility and accessibility of high-quality digitally curated data would be highly beneficial. This would not only decrease risks for irreplaceable physical material (e.g., type specimens), but it would also reduce administrative workloads for curatorial staff and minimize the increasing regulatory complexities associated with border clearance and the exchange of biological samples.

### Data volume

The sheer volume of data held in biological collections (approximately 2 billion–4 billion physical specimens in collections worldwide; Ariño 2010) is beyond human capacity to effectively manage. This situation is clearly exacerbated by the ongoing erosion of relevant capability and funding constraints.

Imaging physical collections is one of the first digitization steps, but this process can be slow because of the care required when physically handling the specimens. Some progress has been made using conveyor belt systems (Tegelberg et al. 2014, Sweeney et al. 2018), and other robotics systems are being developed to speed up these processes (Wührl et al. 2022). However, for digitized specimens to be useful, the metadata for each specimen must be transcribed, and both images and metadata must be checked for errors. Transcriptions and quality control are often performed by curatorial or other trained staff and volunteers or through crowdsourcing platforms such as Notes for Nature or DigiVol (Hill et al. 2012, Ellwood et al. 2018). This is then usually followed by expert verification before being imported into a collection management system, all of which can be a slow and expensive manual process (Hill et al. 2009, Blagoderov et al. 2012). The existing digitization processes cannot keep pace with the volume of specimen data generated, given the need for expert attention at different stages.

Importantly, the types of information that can be obtained from collection specimens is rapidly expanding through the use of new genomics approaches and advanced imaging such as high-resolution, infrared, hyperspectral, and 3D imaging, adding new layers of morphological data, molecular data, and chemical composition data. Furthermore, the increasing amount of field data collected along with physical specimens, such as audio, images, and video, which further document species’ behaviors and interactions, are important sources of information. The broader scientific ecosystem associated with collection specimens, including scientific publications and data sets from molecular analysis, environment, ecology, and so on, is also rapidly increasing, providing new opportunities, as well as challenges, with respect to both data management and collection-based research.

### Data quality and integration

Specimens have been collected over many decades by a wide range of contributors, from professionals to amateur enthusiasts (e.g., citizen scientists). The growing volume of complex data and the diversity of sources require new tools and approaches to support the work of human curators if the collections are to continue to provide high-quality data that are fit for purpose. Evaluating the various biases that can occur in collections is also necessary and should be addressed as this affects overall data quality and research value (Schmidt-Lebuhn et al. 2013, Meineke and Daru 2021).

For specimen metadata already transcribed in databases, there are often significant existing quality issues, including errors, inconsistencies, and omissions in key items such as species and collector names, geolocations, and dates and time of collection (Murphy et al. 2004, Gueta and Carmel 2016, Jin and Yang 2020). Undatabased specimens require the transcription and extraction of metadata items into a database, and these processes can introduce similar errors. Finding and fixing such issues is very time consuming but is of high value (Jin and Yang 2020, Vargas et al. 2023), especially given the need for quality data to inform environmental policy and management approaches.

Keeping up with taxonomic changes is a constant challenge, and the propagation of new information through and among data management systems is problematic. Erroneous taxonomic information can lead to poor conservation decisions (Prie et al. 2012) and public health risk (Carrasco et al. 2016). New tools are needed to help identify and fix errors to ensure the reliability and robustness of the data and metadata (Jin and Yang 2020). This can also help ensure that data are FAIR and trustworthy for researchers and other end users.

The capacity to link collection data to other sources of relevant information, especially across institutions, is in its infancy, but it is increasingly a focus of global efforts. Integration with other data sources is essential when conducting taxonomic and other collection-based research. For example, linking collector names to biographical information and collector field notes can be necessary when determining the correct collector identities and other specimen metadata such as collection locations and dates. The ability to integrate specimen data with genetic information is becoming increasingly important, particularly as the latter becomes more widely available, via databases such as GenBank (Benson et al. 2013, Sayers et al. 2023) and the National Biodiversity DNA Library in Australia (CSIRO 2023).

The digital extended specimen concept builds on this idea (Webster 2018, Gropp 2019, Lendemer et al. 2020, Hardisty et al. 2022) to consider not only genetic data but also the integration of specimen data with other data layers, including environmental, phenological, and morphological. This concept has been further extended to consider other image, sound, and text data (e.g., collectors’ field notes and the scientific literature). Therefore, we need to ensure that linked data sources are also of reliably high quality, enabling connections to be made both explicitly, using standardized identifiers such as ORCIDs for researchers or DOIs for literature, and implicitly, using combinations of data items such as the country, the collector’s name, and the date of collection.

### A global network

Enhancing existing global networks of collections, taxonomic, and curatorial expertise is crucial for accelerating rates of specimen identification and species discovery. Taxonomy evolves constantly, with the identification and discovery of new species, along with changes to taxonomic classifications as new insights emerge. Providing effective and timely alerts for researchers and end users about newly available information on specimens relevant to their areas of interest can be vital for research in areas ranging from the spread of invasive species to human health. Interactive systems that facilitate real-time updates and discussions among taxonomists, curators, and researchers would not only improve the accuracy and currency of global biodiversity databases but also support a more collaborative, inclusive, and productive scientific community. An improved global network would greatly improve collection management and build shared curatorial expertise via a globally consistent and interoperable approach to how we manage and use both physical collections and digital data.

National and international regulations, such as the Nagoya Protocol (Secretariat of the Convention on Biological Diversity 2011), and initiatives such as the CARE Principles for Indigenous Data Governance (CARE for *collective benefit, authority to control, responsibility, and ethics*; Carroll et al. 2020) may place a range of requirements on both physical specimens and digital data that increase workloads on curatorial staff. International loans of physical biological specimens for analysis are particularly problematic as restrictions on the movement of biological samples increase.

## How could human–AI collaboration help?

In the present article, we explain our conceptualization of human–AI collaboration, what its benefits and challenges may be, and our vision for how it may be applied in future biological collection curation, data management, and research. We also introduce the concept of digital curators—AI-based software assistants with their own aptitudes and weaknesses—many of which could be integrated into wider collection use. We believe that the complementary strengths of AI and human abilities can both expand collection capability and lead to a more resilient and productive model of work. Over time, AI-based digital curators will improve to handle more complex data management tasks and to be increasingly trusted as humans learn how to work with them most effectively.

AI-based tools should be designed to complement human experts, which can lead to increased performance compared with human or AI alone (Wilder et al. 2021, Schleiger et al. 2023, McAleese et al. 2024). Successful collaboration between humans and AI necessitates not only interaction but also the ability to adapt as contexts change. This implies some forms of shared understanding or models of situations, states, or contexts (Endsley et al. 2022). A key may be the development of AI-based agents that can interpret and respond to human inputs and humans who can interpret and act on the outputs of the agents. The ability to adapt to changing contexts is crucial, because it allows the human–AI collaboration to respond effectively to new challenges and opportunities and is a defining feature of collective intelligence (Gupta et al. 2023). Contextual awareness involves many aspects, including not only domain and world knowledge but also the recognition of each other's and one's own capabilities and limitations (Kozierok et al. 2021b) that results from shared situational awareness and mutual theory of mind (Gupta et al. 2023, Friston et al. 2024) and that may lead to a vast expansion of collective cognitive capabilities (Gupta et al. 2023).

Our goal is to develop AI-based digital curators with which human curators can interact to augment, extend, and complement their own abilities. Communication would occur using appropriate methods, perhaps using natural language or visual displays when communicating with human team members or with structured or abstract code when communicating with other digital curators or systems. Guidance toward common goals would be provided by the human team members, but guidance (or feedback) may also be instigated by digital curators. Although such a scenario involves implicit workflows, where tasks are performed over time by one or more team members interacting with each other (e.g., improving data quality or knowledge discovery), this is not fixed in advance and adapts to change as necessary.

Although LLMs provide a useful interface for humans, digital curators will also consist of a range of other models and methods as an AI-based system, rather than just an AI model (Zaharia et al. 2024). Digital curators should possess domain-specific knowledge or the ability to acquire it at least semi-independently. This would enable them to use the unique terminologies and procedures within a specific field and to provide relevant and informed assistance. Digital curators should also be capable of updating their knowledge bases as new information becomes available, ensuring their actions are always based on the most accurate data. The incorporation of adaptive learning processes and feedback loops will be important for digital curators to understand the goals and contexts of tasks and allow them to adapt to both changing requirements and their teammates’ capabilities (Kudithipudi et al. 2022, Wang et al. 2023c). Efficient sharing and reuse of knowledge among digital curators may also improve outcomes and scalability and reduce resource use (Soltoggio et al. 2024).

Digital curators should also be able to ask questions to clarify goals and express uncertainty or a lack of knowledge (Cao et al. 2024, Friston et al. 2024, Kim et al. 2024). This ensures that they can better understand the tasks at hand and can prevent misunderstandings and mistakes. This also prompts participants (human or AI) to seek and provide additional information when needed. Finally, digital curators’ capabilities may include searching for and referencing knowledge outside their current domain of expertise and integrating new techniques and technologies (Hope et al. 2023). Although this may seem ambitious, keeping up to date with new techniques would enable digital curators to suggest possible new methods allowing human curators to approve (or not) a particular course of action. These abilities would allow digital curators to provide more comprehensive assistance, drawing on diverse data sources and perspectives, including links to global networks of expertise (Hope et al. 2023). For example, as a step in this direction, GeneGPT (Jin et al. 2023) is a system that uses a novel method for teaching LLMs to use the web application program interfaces (APIs) of the National Center for Biotechnology Information to answer genomics questions.

Collaboration between human curators and AI-based digital curators has the potential to extract more knowledge from specimen-based data than either could alone and will play a key role in managing, curating, and analyzing these large, diverse, and expanding knowledge bases. In a similar way to providing training to human assistants so that they can improve their expertise and value, so too will we provide assistance to digital curators to ensure they can fulfil their roles. Human–AI collaboration would benefit human curators directly through assisting with tasks such as trait extraction and species identification and indirectly by freeing them to do other work that either cannot be delegated to digital curators or that they prefer to do themselves.

## Toward collaborative AI

There are already many examples of AI models and methods being applied to biological specimens and biodiversity in specific contexts. These include computer vision applications such as species recognition (Favret and Sieracki 2016, Unger et al. 2016, Norouzzadeh et al. 2018), trait extraction (Younis et al. 2018, Pearson et al. 2020, Weaver et al. 2020), and transcription using OCR (optical character recognition) from specimen images (Barber et al. 2013, Alzuru et al. 2019, Dupont and Price 2019); clustering techniques for anomaly detection and standardization within data sets (Hill 2016); natural language processing methods for translation and named entity recognition (Alzuru et al. 2020, Owen et al. 2020, Abdelmageed et al. 2022); species detection (Walters et al. 2012, Xie et al. 2016, Stowell and Sueur 2020, Kahl et al. 2021); and soundscape analysis from audio samples (Blumstein et al. 2011, Stowell and Sueur 2020, Lin et al. 2023).

For most of these applications and technologies, the interaction between human and AI is limited to the human providing training source material while iteratively developing and evaluating the AI or predictive model before applying it to the data of interest and evaluating the results. The interaction is effectively limited to using AI methods to assist in curation tasks, without any real collaboration involving shared goals or contextual understanding. Examples that involve more prolonged human–AI collaborations applied to collections curation and management are lacking.

More advanced examples of human–AI collaboration come from other domains, most recently resulting from advances in neural network models using generative pretrained transformers (GPTs; Vaswani et al. 2017, Radford et al. 2018). LLMs using GPTs are predictive models trained on extensive collections of textual data. They use statistical methods to predict the next word or part of a word when answering a query. Although they demonstrate useful capabilities in multiple areas (Microsoft Research AI4Science and Microsoft Azure Quantum 2023), especially when they are large scale or fine-tuned for particular tasks, for the purposes of collaborative work, a key aspect is the context that is supplied to them. The provision of context provides additional information to a query. This can act as a form of memory when used to supply a history of interactions, it could provide more information about the aim of the current task, or both (Majumder et al. 2023). This may change and depends on how the interaction with the model is designed. Both fine-tuning to a knowledge domain and enhancing context provision will be important for adapting these types of systems to collection curation and management tasks. For example, when biodiversity researchers use collection specimen data, the specific context of the research question and aims will determine whether particular specimen data are suitable for the intended purpose or not (Heberling 2022).

Other domains also provide examples of concrete steps toward human–AI collaborative work, as well as some of the challenges. Research in medical clinical imaging provides examples where AI and human working together provide better results than either working individually (Reverberi et al. 2022, Dvijotham et al. 2023) but also cautionary examples, such as where human–AI collaboration has not worked successfully (Agarwal et al. 2023). These contrasting results point to important considerations, including variability in human performance and possible system design characteristics that influence interactions and outcomes (Cabitza et al. 2021, Morrison et al. 2023).

LLMs can enable better use of natural language queries in an interactive manner, which may provide a better user experience and reduce the barrier of learning system-specific commands, language, or coding skills (Brown et al. 2020). The recent development of tools such as ChatGPT and others demonstrate how iterative refinement of queries can enable better progress toward a goal and illustrates the importance of sharing past context for mutual understanding (Microsoft Research AI4Science and Microsoft Azure Quantum 2023, Lin 2024). However, this relies on the accuracy and validity of responses, which can sometimes be questionable (Roller et al. 2021, Ji et al. 2023, Nezhurina et al. 2024). There are a variety of methods to improve LLM results, such as parameter modification, improving contextual information using a variety of techniques and evaluating results using other models (Shuster et al. 2021, Wei et al. 2022, Liu et al. 2023, Zheng et al. 2023). Easy-to-use prompting techniques—that is, methods of structuring queries to LLMs, such as zero shot, few shot, chain of thought, and plan and solve—have shown promise in improving the precision and relevance of LLM outputs (Liu et al. 2023, Wang et al. 2023b, Lin 2024). Schulhoff and colleagues (2024) provided a comprehensive overview of current prompting techniques, from text-based to multimodal methods.

Github's CoPilot Chat (https://docs.github.com/en/copilot/github-copilot-chat) and CoPilot Workspace (https://githubnext.com/projects/copilot-workspace) provide applied examples of domain-specific AI-based assistants, in these cases for software development, which significantly improve productivity, including more benefits for older and less experienced participants (Peng et al. 2023). PathChat is a vision–language generalist AI assistant for human pathology that combines a foundational vision encoder with a pretrained LLM with the system fine-tuned on domain-specific data (Lu et al. 2024b). In addition to fine-tuned models, domain-specific agents can be augmented with external tools, such as web search and retrieval augmented generation (Lewis et al. 2021), which can provide extra context from domain-specific knowledge bases to enable better performance using natural language on knowledge intensive tasks. Other recent applications using LLM-based agents, such as AutoGPT and GPT Engineer, offer interactive and semiautonomous capabilities, including additional abilities such as automatic prompt generation and interactive clarification of goals and intentions. Combining these capabilities with multiple and more capable multimodal AI models, such as GPT-4o (OpenAI 2023a), Gemini (Anil et al. 2023), and Claude (Anthropic 2024), along with other specialized and hybrid models and systems, suggests very powerful approaches will soon be possible (Miao et al. 2024), including for the diverse range of possible AI-assisted tasks in biological collections. The increasing demand for high-quality, curated digital data from collections worldwide is a major driver for such specialized models.

Human–AI collaboration in biological collections offers significant potential but also presents several challenges. The development and use of AI models often requires substantial computational and human resources, which can be prohibitively expensive for many collections. Concerns about the reliability, transparency, and ethical implications of AI models persist, particularly when the sources of training data are unknown, potentially leading to various biases and errors (Ntoutsi et al. 2020, Mehrabi et al. 2021, Gallegos et al. 2024). To address these issues, explainable AI and other interpretability and mitigation methods should be considered for the evaluation of and building trust in AI systems (Adadi and Berrada 2018, Brundage et al. 2020, Ali et al. 2023, Gallegos et al. 2024).

Although both humans and AI can make mistakes, potentially compounding errors in collaborative systems (Guo et al. 2024), research has shown that human performance improves when working alongside AI (Grønsund and Aanestad 2020, Hemmer et al. 2024) and that imperfect AI system outputs can still be useful (Weisz et al. 2022). The beneficial effect of diversity (Fischer 2023) extends to AI systems themselves. Architectures, such as the mixture of experts, leverage internal diversity by dynamically routing inputs to specialized subnetworks within a single model, potentially improving performance and efficiency (Shazeer et al. 2017, Artetxe et al. 2022). Similarly, ensemble methods, which leverage the collective strength of multiple models or agents, produce better results than single model systems (Guralnick et al. 2024, Wang et al. 2024a). These approaches are, perhaps, small steps toward emulating the concept of collective intelligence (Arima 2021) or an ecosystem of intelligence (Friston et al. 2024) observed in biological systems across various scales, from cellular networks to ecosystems, where diverse entities collaborate to solve complex problems and adapt to changing environments (McMillen and Levin 2024).

To maximize the potential of human–AI collaboration within collections, it is essential to design systems that promote and enhance human curatorial skills, knowledge, and satisfaction. Kozierok and colleagues (2021a) proposed key characteristics defining effective human–machine collaboration, including mutual contribution of meaningful content, context awareness, robustness, and consistent and satisfying human engagement. These properties can serve as a useful framework for evaluating and improving future collaborative systems in biological collections and other domains.

## What could the future look like

**Figure 1.**
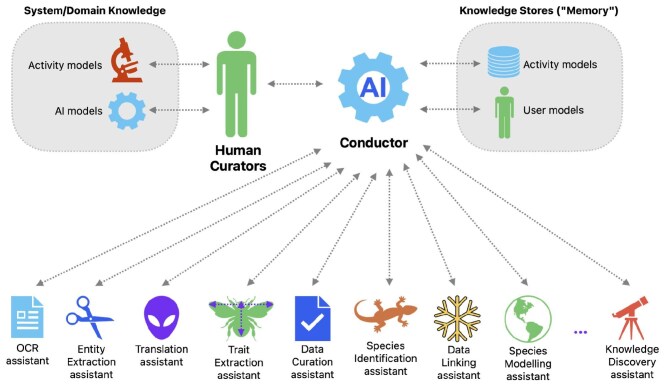
One model for future collection curation using diverse digital curators. Human curators supply domain-specific knowledge and guidance about activities and interactively improve their understanding of the system and AI models’ capabilities. The top-level digital curators (or conductors) could access background models or information on users and activities or tasks to better understand the context of current queries and actions and therefore be able to better collaborate with the human curators and adapt their actions (e.g., choosing which subset of digital assistants are required) to these contexts. The bidirectional arrows indicate that communication occurs in both directions. Other possible models incorporating more flexible communication among all these entities may also be desirable, where user or activity models might also be accessed by any digital curator.

Digital curators could take many forms, including conversational assistants able to use LLMs with access to collections-specific knowledge. This will enable extended discussions and explorations using prior context of past interactions as well as knowledge of current task-specific goals, which may be interactively clarified during the conversation between human and digital curators (Ross et al. 2023). Numerous specialized curatorial assistants are likely to be available (OECD 2023), with appropriate selection of which to use for any particular task made by a human or digital curator as needed. Figure 1 illustrates one of many possibilities for collection curation collaboration with a range of specialized digital curators interacting with human curators through an orchestrator or manager digital curator.

For example, some digital curators might specialize in specific taxonomic groups, serving as a reference point by keeping up to date with taxonomic changes within these groups, automatically scanning and parsing literature libraries for relevant information and updating their own (or shared) memory or data store. Communication with other curators, both digital and human, could occur automatically, with subsequent actions depending on the context. One digital curator with human-language communication abilities might notify human curators of potential taxonomic changes that need their attention or require their expertise to confirm they are valid, while at the same time relevant reference material and data gathered by another digital assistant could then be presented in a usable form to the human curator.

Digital curators promise to augment a range of tasks, significantly accelerating and enriching curatorial, management, and research processes, including information extraction, where they could efficiently mine valuable data from various digital formats (Dagdelen et al. 2024, Gougherty and Clipp 2024). This may include extracting metadata and traits from specimen images and literature (Weaver et al. 2020, Abdelmageed et al. 2022) or deciphering behavioral phenotypes from multimedia sources (Webster 2018). Digital curators could also find errors and anomalies in existing data sets and suggest and verify corrections. This would enhance the quality and reliability of research data, forming a crucial foundation for subsequent analysis. By intelligently connecting related and disparate data sources, digital curators could contribute to the development of the digital extended specimen network, fostering a more holistic understanding of biodiversity. This may contribute to scientific discovery through discovering hidden patterns and generating novel hypotheses for further investigation (Liekens et al. 2011, Hope et al. 2023, Wang 2023).

They could identify known species from specimen images and audio (Stowell et al. 2018, Wäldchen and Mäder 2018, Little et al. 2020), as well as flag potentially unknown species or interesting specimens (Tuia et al. 2022, Lu et al. 2024c). Other digital assistants could provide support for planning, testing, and evaluating hypotheses or suggesting suitable methods to do so. In addition, they could help in discovering knowledge gaps and identifying the best methods to fill them. By collating, analyzing, and summarizing scientific literature and data sets, they could provide relevant information for the task at hand (Microsoft Research AI4Science and Microsoft Azure Quantum 2023, Spillias et al. 2024).

Digital curators could identify subject matter experts and coordinate communications with them when more expertise is required. This would allow researchers to access the necessary knowledge and guidance from other human experts in their field. By combining their natural language skills with memory stores, such as vector databases or knowledge graphs, these assistants would have the ability to dynamically interact with various knowledge sources and provide valuable insights and support. Overall, the possible uses of AI-based digital curators are vast and diverse. They have the potential to revolutionize the way collection curators and researchers work by providing them with assistance in various tasks, from data extraction and analysis to hypothesis generation and expert coordination. With their contextual understanding and communication capabilities, these assistants could enhance productivity and efficiency in scientific research and knowledge discovery.

### Example scenarios

Following are three of many possible examples, where digital curators could offer great assistance to collection curators and researchers using collections data. The third scenario illustrates one possible use of the architecture presented in figure 1 to use a set of digital curators as skilled assistants for scientific research.

A central part of curation is ensuring that identifications remain up to date. This task presents several challenges, such as staying informed about newly published taxonomic tools, revisions, and monographs (Fawcett et al. 2022); managing the identification of newly accessioned specimens; and updating names that have changed because of nomenclatural rules even if species delimitation stays the same (Grenié et al. 2023). The curators’ work could be significantly enhanced through automated systems incorporating alerts, literature mining agents, computer vision–aided identification tools, and database monitoring scripts. A digital curator could alert curators to new or newly digitized taxonomic treatments, enabling reidentification efforts. An LLM-based digital curator could summarize species descriptions into user-friendly formats, such as tables, whereas another agent could query the specimen database, identifying potentially affected specimens, including those misidentified under outdated synonyms or related genera. A first step toward such systems are models that recognize taxa in ecological literature (Agosti et al. 2024), allowing the automatic connection of additional trait data to species (Le Guillarme and Thuiller 2023). Day-to-day curation could be assisted by a digital curator that brings to the curator's attention outlier specimens that do not match the others filed under the same name and maybe even suggests which ones they do match visually and in terms of geographic occurrence, flowering time, or other metadata.

The data from collection specimens are used in various types of research (Cicero et al. 2017, Monfils et al. 2022). These include spatial biodiversity analysis, such as identifying conservation priority areas on the basis of diversity hotspots (Mishler 2023), studying phenology (e.g., flowering and fruiting times) and its response to global changes (Jones and Daehler 2018), and detecting collecting biases (Daru et al. 2018). These analyses depend on accurate data, which researchers must often laboriously clean after extracting it from databases or aggregators such as the ALA or the GBIF (Chapman 2005, Hill et al. 2010). Unfortunately, this cleaning process is repeated independently each time, with little standardization, and corrections rarely make their way back to the original collections. Digital curators could help by continuously improving data quality, flagging geographic, temporal, and other inconsistencies for their human curators; by suggesting missing data that can be inferred from context, such as geographic coordinates; and by updating databases efficiently with bulk corrections, reducing human error—while ensuring human curators remain involved to prevent AI overreach in areas of uncertainty.

**Figure 2.**
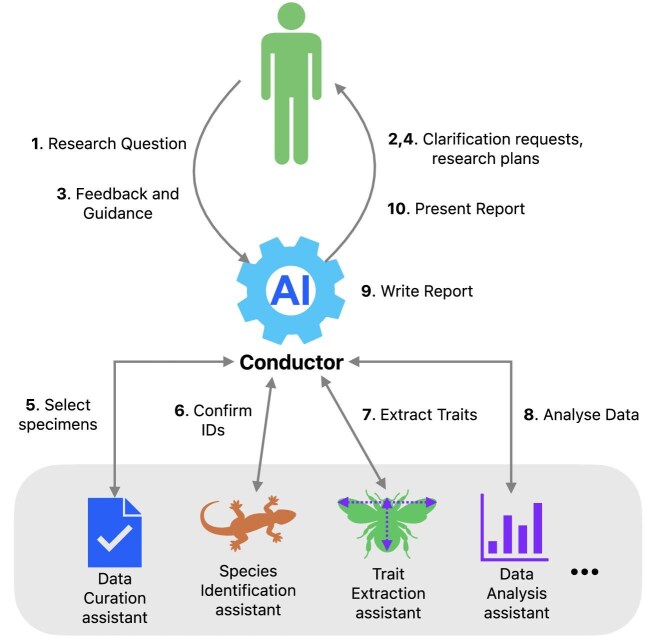
An example scenario, using the architecture from figure 1, showing a possible interactive research workflow looking at climate change disruption to mutualistic relationships. The process starts with a research question posed by a human researcher. The AI-based conductor may seek clarification and guidance from the researcher while developing a research plan. The conductor then coordinates the workflow by engaging specific assistants to select suitable specimens using the data curation assistant, confirm the specimens are accurately identified with the species identification assistant, extract trait information for each using the trait extraction assistant, and then analyse the data using the data analysis assistant. The results are sent back to the conductor, which proceeds to write a report that is returned to the researcher. The diagram illustrates a possible interactive flow among the researcher, the conductor, and the digital assistants.

Imagine that a researcher wants to study how climate change may cause temporal mismatches between flowering plants and their pollinators, potentially leading to disruptions in mutualistic relationships (see figure 2). Posing this question to their personal digital curator (or conductor) should result in a series of questions and answers to clarify the requirements and context of the research. The conductor, using its knowledge of the abilities of other available digital curators, then works iteratively and interactively with the researcher to develop a research plan that is then actioned. The conductor would then, in consultation with the researcher, oversee the plans, actions, and responses of the various digital curators called on to contribute to the work. For example, the data curation assistant may be called on to select suitable collection specimen records from around the world. These may be passed to the species identification assistant to confirm the accuracy of species identification before then being passed to the trait extraction assistant if needed and so on. Throughout the process, the researcher provides feedback and guidance, intervening and modifying plans as desired.

## How do we get there

In this section, we propose some actions to help unlock the value within biological collections using human–AI collaboration, using the strengths, and mitigating the weaknesses of each. First, we need to ensure that specimen data are of sufficient quality to promote trustworthy and efficient reuse and sharing and interoperability, following the FAIR model. This is critical in several ways: As data become more available globally, they will be used more widely; avoiding repeated local data cleaning tasks ensures faster, more trustworthy, and repeatable research. AI models will be trained on these data, so ensuring a source's data quality is vital for high-quality models, particularly because trained models are shared widely after development. The effects of both high- and low-quality data will be multiplied as information is increasingly propagated across the global network and used in integrated applications.

To do this at the scale required, we can use digital curators to process images, extract and verify specimen data, and link these data to other data sets as we move toward the realization of the digital extended specimen concept. Digital curators can also assist with cleaning up existing data sets by identifying outliers and anomalies that, if not able to be corrected automatically or semiautomatically, are then brought to the attention of human curators. Achieving this may require the development of knowledge bases incorporating relevant scientific literature and new methods of interacting with them using AI models and tools, and continued progress toward agreed standards for data collection, storage, and sharing.

Realizing this within global collections will depend on leveraging the experience, models, code and artifacts from other industries, and the continued sharing of experience and artifacts within the collections community (Greeff et al. 2022). The platforms Hugging Face (www.huggingface.co) and Kaggle (www.kaggle.com) are two examples where a range of AI-related resources, such as models, code and data sets, are shared, allowing everyone to learn and make use of them. There are also numerous ways to download and use LLMs on local hardware such as PCs without requiring programming expertise, including GPT4All (www.nomic.ai/gpt4all), LM Studio (https://lmstudio.ai), and Jan (https://jan.ai). Other specialized AI models, such as BioCLIP for species identification (Stevens et al. 2023), can also be downloaded and used locally or accessed via APIs if local hardware or software is lacking. Making effective use of these tools may require learning some new skills, however, including the development of human–AI collaborative interaction and evaluation techniques that ensure our models and methods of using them provide accurate and trustworthy results (Caldwell et al. 2022, McAleese et al. 2024).

We also need to make further progress toward specialized assistants that may share many general-purpose capabilities but with their own contextual knowledge that guides their actions, in much the same way humans rely on their own knowledge and experience to guide their own actions. Some of these general-purpose capabilities may include adaptive communication and behavior depending on context, proactive suggestions of possible actions, collaborative problem solving, and conflict resolution.

To realize a scenario such as described in figure 2, digital curators must have contextual understanding and communication capabilities. Contextual understanding implies some forms of memory, which consists of long-term memory in the form of their base AI model or models, as well as medium- and short-term stores that provide important historical and contextual information about both tasks and users to guide their current actions (Lin 2024, Liu et al. 2024). This is currently achieved by combining LLMs with memory stores such as vector databases or knowledge graphs. LLMs can provide natural language skills that are useful for a large variety of tasks, whereas memory stores are more dynamic and provide vital task-specific contextual information. Enabling a digital curator to dynamically interact with LLMs and other knowledge sources provides great power, with the human curator providing the overall aims for these actions, along with the ability to interrupt and redirect as needed.

Recent progress toward customized agents using the power of LLMs together with local, context specific knowledge bases can be seen with the recent availability of so-called GPTs (OpenAI 2023b) and similar frameworks. Other recent platforms that provide customizable agents along with data stores and the use of a variety of AI models include the LangChain system (www.langchain.com), AutoGen Studio (Dibia et al. 2023, Wu et al. 2023), and CrewAI (www.crewai.com). These provide capabilities for agents to interact or communicate with humans, as well as with other agents, in addition to using both prepackaged and bespoke software tools such as internet search, API and database access, and code execution. An important feature of these customizable multiagent systems (Huang et al. 2024, Wang et al. 2024a) is providing a means of evaluating the agents’ (or in our context, digital curators’) responses (Wang et al. 2023a), to interactively (i.e., human controlled) or automatically (by other agents) review and revise the responses, directions, or context that have been provided, in much the same way that we constantly evaluate and clarify common understanding in human conversations. A simple audit trail of plans, decisions, and actions taken by digital and human curators may also be useful for review, evaluation, and improving performance, assisted by other AI-based tools—for example, the Claude insights and observations system by Anthropic (Tamkin et al. 2024).

Box 1. **Glossary of AI-related terms.**

**AI (artificial intelligence).** The development of computer systems capable of performing tasks that typically require human-level cognition, such as learning, reasoning, and problem-solving.

**Agentic systems.** AI systems designed to operate independently within a defined environment, making decisions and taking actions towards specific goals.

**API (application programming interface).** A defined set of rules and protocols that enable different software applications to communicate with one another.

**Computer vision.** A subfield of AI that empowers computer systems to analyze and interpret digital images and videos, extracting meaningful information (e.g., identifying shapes, patterns, textures, and objects) for tasks such as species recognition.

**Generative pretrained transformer.** A neural network architecture specialized for language tasks, utilizing transformer technology and pretraining on large text data sets to generate contextually relevant content.

**Knowledge graph.** A structured network of interconnected entities and their relationships, organized to facilitate searching, inference, and reasoning across data.

**Large language model (LLM).** An AI model trained on a huge volume of text data, enabling them to understand, generate, and manipulate human language. LLMs capture complex patterns in language, making them versatile tools for tasks such as text summary, translation, and answering questions.

**Machine learning.** A core AI approach where algorithms improve their performance by learning from data rather than explicit programming, enabling pattern recognition and prediction in complex data sets.

**Multimodal AI.** AI systems that process and integrate data from different sources, such as text, images, and audio, enabling a more comprehensive understanding of complex information.

**Neural network.** A computational model inspired by the structure and function of biological neural networks, consisting of interconnected nodes (neurons) organized in layers that process and transform data for various machine learning and AI tasks.

**Natural language processing.** A subfield of AI focused on enabling computers to understand, interpret, and generate human language (text or speech), facilitating communication between humans and computers, and enabling the analysis of textual data.

**OCR (optical character recognition).** Uses AI models to extract text from images and convert it into machine-readable text, streamlining the extraction of data from scanned documents.

**Vector database.** A database that stores data in the form of numerical vectors (mathematical representations), allowing for efficient searching and retrieval of information on the basis of the similarity or relationships between vectors.

**XAI (explainable AI).** A set of techniques aimed at making AI decision-making processes more transparent and interpretable for humans. XAI aims to reveal the underlying logic behind AI outcomes, enhancing trust, validation, and accountability in AI systems.

There are a number of architectures for multiagent systems such as RAISE, MetaGPT, and AutoGen (Hong et al. 2023, Wu et al. 2023, Liu et al. 2024), that extend single-agent architectures. These enable dynamic teams of agents to be created and deployed for certain tasks, depending on their roles and skills, with planning, execution, and evaluation occurring iteratively. Key features include clear leadership roles, dynamic team construction, and effective information sharing (Masterman et al. 2024). Guo and colleagues (2024) showed that these teams are most effective when the leader is human. In the future, lifelong or continual learning algorithms will assist the development of a digital curator collective where the exchange of knowledge among agents enables integrating knowledge learned at different times, on different tasks, and by different agents (Soltoggio et al. 2024, Wang et al. 2024b). Recent examples of multiagent systems from other domains include ChemCrow for organic synthesis, drug discovery, and materials design (Bran et al. 2023); ProtAgents, which combines physics simulations and machine learning for protein discovery (Ghafarollahi and Buehler 2024); and the AI Scientist, which performs ideation, literature search, experiment planning, experiment iterations, manuscript writing, and peer reviewing to produce papers in the machine learning field (Lu et al. 2024a).

Although the technical challenges attract much attention, the environmental, social, and economic implications of deploying such systems are also important. Ethical considerations include the responsible design and implementation of AI agents that respect human values, mitigate biases, and promote fairness (Gallegos et al. 2024, Lu et al. 2024a). Addressing the potential displacement of jobs may require proactive strategies for workforce transition, including programs that combine foundations in biology, taxonomy, and systematics with core data and AI-related skills (National Research Council of the National Academies 2015, Monfils et al. 2022). We should also consider environmental sustainability and strive for efficient processes, models, and code (Wu et al. 2022, Samsi et al. 2023).

The global collections community is developing key foundational aspects of integrated digital collections, such as persistent digital identifiers for specimens and other entities (Güntsch et al. 2017, Nelson et al. 2018, Addink and Theocharides 2024), databases containing data sets and literature along with associated metadata and accessible through APIs (Bánki et al. 2023, Penev et al. 2024), and initial biodiversity and collections knowledge graphs (Page 2019, Dimitrova et al. 2021, 2023). By leveraging these foundations to create specialized AI-based agents for biological collections and integrating them with more general-purpose collaborative agents, we could facilitate communication between digital and human curators and enhance human curatorial work and research. Sharing digital curators and other artifacts and our experiences with them within the collections community should enable wider and more effective use. This approach will enable us to build robust human–AI collaborative systems to extract more value and knowledge from our specimen collections and better appreciate the important role collections play in understanding our rapidly changing world and addressing our global challenges.

## Conclusions

In the future, we envisage fully digitized, globally accessible natural history collections that are integrated with other data sources, providing a comprehensive view of biodiversity—a global biodiversity semantic web. This will be enabled by continued global progress on collections digitization and movement toward the global digital extended specimen network. Multiple digital curators will have been developed and deployed, with the ability to fill diverse capability gaps (or provide novel capabilities), enabling us to augment and extend our combined abilities to examine problems that we have not yet faced and to discover new knowledge.

## Author Biography

Alan Stenhouse (alan.stenhouse@csiro.au), Nicole Fisher, Brendan Lepschi, Alexander Schmidt-Lebuhn, Juanita Rodriguez, Federica Turco, and Peter H. Thrall are affiliated with the National Collections and Marine Infrastructure Research Unit at the Commonwealth Scientific and Industrial Research Organisation (CSIRO), in Canberra, Australia. Brendan Lepschi is affiliated with Parks Australia, in Canberra, Australia. Brendan Lepschi and Alexander Schmidt-Lebuhn have additional affiliations with the Centre for Australian National Biodiversity Research, also in Canberra. Andrew Reeson and Cécile Paris are affiliated with CSIRO's Data61 Research Unit
